# Research Progress of circRNAs in Glioblastoma

**DOI:** 10.3389/fcell.2021.791892

**Published:** 2021-11-22

**Authors:** Xu Guo, Haozhe Piao

**Affiliations:** Department of Neurosurgery, Liaoning Cancer Hospital and Institute, Cancer Hospital of China Medical University, Shenyang, China

**Keywords:** circRNAs, non-coding RNAs, biomarker, glioblastoma, miRNAs sponge

## Abstract

Circular RNAs (circRNAs) are a class of single-stranded covalently closed non-coding RNAs without a 5′ cap structure or 3′ terminal poly (A) tail, which are expressed in a variety of tissues and cells with conserved, stable and specific characteristics. Glioblastoma (GBM) is the most aggressive and lethal tumor in the central nervous system, characterized by high recurrence and mortality rates. The specific expression of circRNAs in GBM has demonstrated their potential to become new biomarkers for the development of GBM. The specific expression of circRNAs in GBM has shown their potential as new biomarkers for GBM cell proliferation, apoptosis, migration and invasion, which provides new ideas for GBM treatment. In this paper, we will review the biological properties and functions of circRNAs and their biological roles and clinical applications in GBM.

## Introduction

Glioblastoma (GBM) is one of the most malignant primary brain tumors in adults, characterized by an expansile and infiltrative growth pattern (Jackson et al., 2019; Tan et al., 2020; McKinnon et al., 2021). According to the World Health Organization (WHO) classification for central nervous system (CNS) tumors, GBM is classified as the highest grade IV (Broekman et al., 2018; Caragher et al., 2018). Currently, the standard of therapy for GBM is surgical resection with maximum safety followed by concurrent radiotherapy and adjuvant chemotherapy (Alifieris and Trafalis, 2015; Karachi et al., 2018; Choi et al., 2019; Geraldo et al., 2019). However, the efficacy of this regimen is limited, and the median survival of patients after treatment is only 15 months (Touat et al., 2017; Lim et al., 2018; Balca-Silva et al., 2019). To better treat GBM patients and improve their survival time and quality of life remains a huge challenge. Therefore, the study of mechanisms regulating the malignant progression of GBM and the explore for early GBM biomarkers are important for the early diagnosis, treatment and prognosis of GBM.

Circular RNAs (circRNAs) are covalently contiguous closed loops without 5′ and 3′ ends, and are structurally more stable than linear RNAs and less susceptible to degradation by nucleic acid exonucleases (Jakobi and Dieterich, 2019; Huang and Zhu, 2021). Initially circRNAs were thought to be products of missplicing or intermediates escaping from the lasso structure of introns (Chen and Huang, 2018; Tsitsipatis and Gorospe, 2021). With the widespread use of transcriptome sequencing technologies, numerous studies have identified circRNAs as a class of endogenous, numerous molecules that are stably present in mammalian cells with certain organizational, temporal, and disease properties and are no longer considered a class of RNA molecules with no role in the human body (Han et al., 2018; Wu J. et al., 2021; Choudhary et al., 2021). CircRNAs present in mammalian cells, there are over 400 circRNAs in normal humans whose abnormal expression can induce tumorigenesis (Ebbesen et al., 2017; Zhou et al., 2020; Zhang et al., 2021a; Shao et al., 2021).

Studies have showed that circRNAs are involved in the occurrence and development of GBM due to their highly stable ring structure, high abundance in cancer tissues and relative tissue specificity, and their altered expression is expected to become a new marker for early diagnosis and prognostic assessment of GBM or a new target for effective treatment. This review summarized the research progress of circRNAs in GBM in recent years, including the mechanism of circRNAs occurrence, function and application research in GBM.

## Biogenesis and Classification of Circular RNAs

CircRNAs were first identified in RNA viruses in 1976, and in 1979, Hsu et al. discovered a ring-like molecule with covalently linked 3 and 5′ ends in Hela cells by electron microscopy (Hsu and Coca-Prados, 1979). Because of its special structure, it was often ignored as an abnormal shear by-product. It was not until 1993 that the existence of this structurally unique closed-loop noncoding RNA was confirmed in humans (Farooqi et al., 2021; Wang X. et al., 2021; Lauretti et al., 2021; Sempere et al., 2021). In recent years, with the widespread application of transcriptomic gene sequencing and biophysical techniques, the biological functions of circRNAs and their roles in the development of human diseases are gradually being better understood with the help of high-throughput sequencing technologies. The circRNAs are mainly formed by processing protein-coding genes by RNA polymerase II(Ashwal-Fluss et al., 2014; Li et al., 2019; Ali et al., 2021; Sinha et al., 2021). Meanwhile circRNAs biosynthesis is mediated by RNA binding proteins, intron pair driven and lasso driven, and thus has an important role in regulating adjacent splice sites and promoting circular biosynthesis (Wu et al., 2021a; van Zonneveld et al., 2021; Zhao et al., 2021). Currently circRNAs have also been shown to have many characteristics. Diversity and abundance, circRNAs are widely found in eukaryotic cells and are very diverse (Glazar et al., 2014; Liu et al., 2020; Han et al., 2021). Stability, unlike linear RNA, circRNAs is a single-stranded, covalent closed-loop structure without a 5′ cap structure and a 3′ terminal ploy(A) tail. This structure may protect it from degradation by RNA exonuclease (RNAser) and thus has higher stability than linear RNA (Suzuki and Tsukahara, 2014; Di Timoteo et al., 2020; Wang et al., 2021h). Conservative, circRNAs is highly conserved across species (AbouHaidar et al., 2014; Mao et al., 2021; Meyer et al., 2021). Specificity, mainly in terms of cell type specificity and tissue specificity (Shang et al., 2019; Gokool et al., 2020; Huang and Zhu, 2021).

**FIGURE 1:**
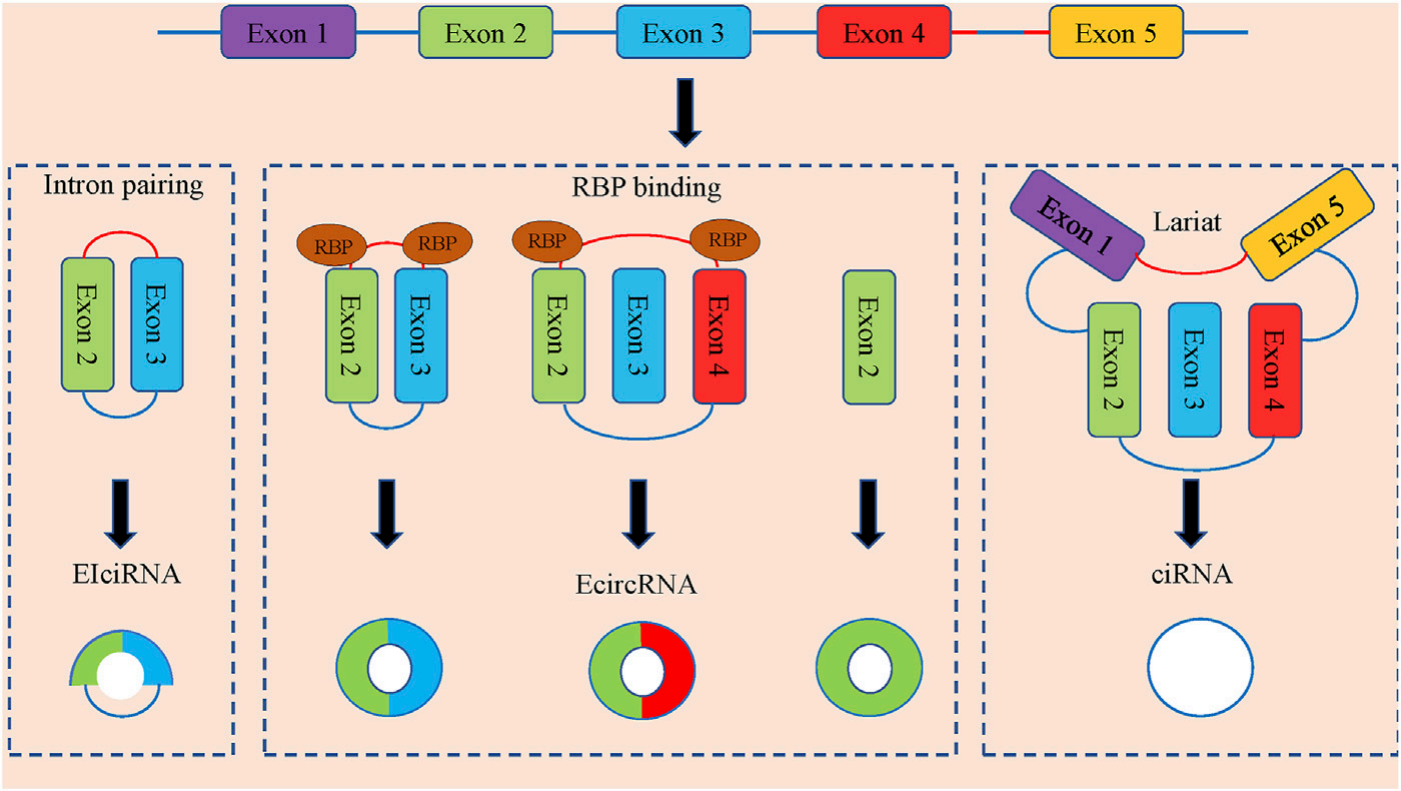
The formation and classification of circRNAs. CircRNAs are commonly divided into EIciRNAs, EcircRNAs and ciRNAs in accordance with their components, which were derived from exons and introns, and both of them in pre-mRNAs, respectively. Latant ecircRNAs can be generated from one pre-mRNA via alternative splicing. The red segment and blue segment between Exon 4 and Exon 5 represented a 7-nt GU-rich motif near the 5′ splice site and an 11-nt C-rich motif at the branchpoint site, respectively, which promoted the generation of ciRNAs.

CircRNAs can be divided into three categories according to the composition of splicing (Figure 1). Exonic circRNAs (E-circRNAs) are composed of backward-sheared exons, intron circRNAs (ciRNAs) are composed of introns only, exon-intron circRNAs (eiciRNAs). Contains both exons and introns. Jeck et al., (Jeck et al., 2013), proposed two different exon cyclization modes, lariat-driven cyclization mode and intron pairing-driven cyclization mode.

## Biological Functions of Circular RNAs

**FIGURE 2:**
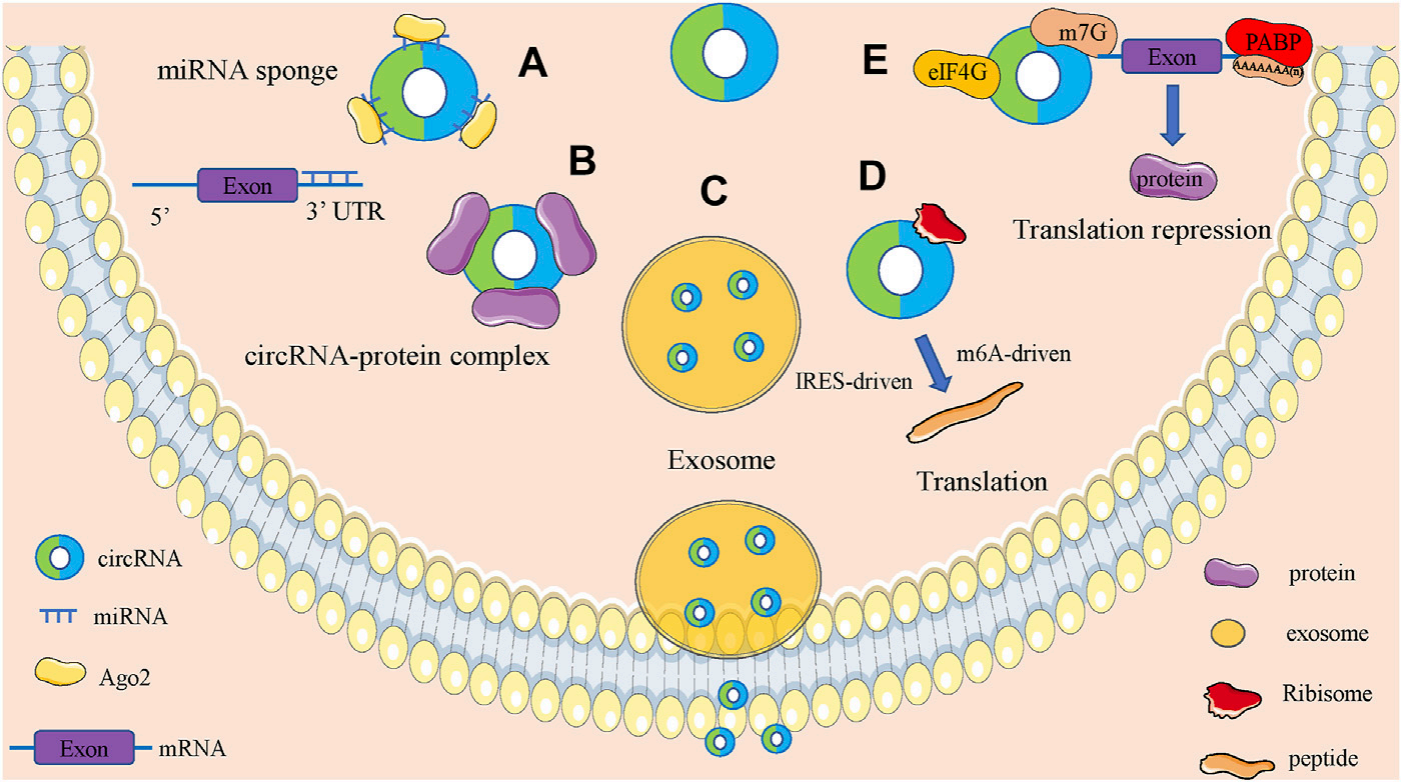
Biological function of circRNAs. **(A)**. CircRNAs can act as miRNA sponges to regulate the expression of downstream genes. **(B)**. CircRNAs can bind to RNA binding protein and regulate parental gene expression. **(C)**. Some circRNAs can be carried by exosomes and involved in the process of cell-cell communication. **(D)**. CircRNAs can encode proteins based on IRES-driven and m6A-driven models. **(E)**. CircRNAs can bind with proteins to establish circRNA-protein complexes and alter the functions of some proteins.

CircRNAs are functionally diverse and are often found to function as microRNAs (miRNAs) sponges because they are rich in miRNAs binding sites. In addition, circRNAs also have roles in regulating parent gene expression, regulating parent gene selective splicing, translating protein functions and participating in intercellular communication by entering exosomes (Figure 2).

### MicroRNAs Sponge

Through miRNAs response elements, non-coding RNAs and coding RNAs form a large-scale regulatory network in the transcriptome. MiRNAs are negative regulators of gene expression, reducing the stability of target genes or limiting their translational function (Salmena et al., 2011; Su and Lv, 2020; Shen et al., 2021). CircRNAs are rich in miRNAs binding sites and competitively repress transcriptional regulation of miRNAs, a new class of highly expressed and stable ceRNAs (Shi et al., 2013). The cyclic RNA hsa_circ_0043280 can reduce PAQR3 levels by competitively absorbing miR-203a-3p and blocking miR-203a-3p, and can function as a tumor suppressor to inhibit tumor growth and metastasis in cervical cancer (Zhang et al., 2021b). Hsa_circ_0006349 promotes MKP1 expression through uptake of miR-98, which enhances proliferation and glycolysis of non-small-cell lung cancer (NSCLC) cells and promotes malignant progression of tumors (Qin et al., 2021). Circ-PPP1CB is downregulated in bladder cancer and negatively correlates with clinical stage and histological grade. Circ-PPP1CB regulates cell growth, metastasis and epithelial mesenchymal transition (EMT) by interacting with the miR-1307-3p/SMG1 axis (Wang F. et al., 2021). Circ-EYA3 is elevated in pancreatic ductal adenocarcinoma (PDAC) tissues and cells, and higher levels of circ-EYA3 are significantly associated with poorer prognosis in PDAC patients. Circ-EYA3 can enhance c-Myc expression by acting as an endogenous miR-1294 sponge, which in turn promotes ATP synthesis to increase energy production and promote malignant progression of PDAC (Rong et al., 2021a).

### Interaction with RNA-Binding Proteins

CircRNAs can alter splicing patterns or RNA stability by binding to RNA binding protein (RBP) (Huang A. et al., 2020; Zhou et al., 2020; He A. T. et al., 2021; Li J. et al., 2021). Muscleblind (MBL) is a muscleblind-like 1 (mbnll) MBL promotes the production of circmbl, which has a specific MBL binding site, and circmbl has a strong direct interaction with MBL protein (Kristensen et al., 2019). Circ-Mbl regulates MBL protein levels and reduces its own mrna production by promoting circmbl production when MBL is in excess. Circmbl can also eliminate excess MBL by binding to MBL (Ashwal-Fluss et al., 2014). Circrna can facilitate the interaction between DNA, RNA, and RBP to perform biological functions by binding to related proteins (Qi et al., 2021). Circ-RNF13 prolongs the half-life of SUMO2 by binding to the 3′ untranslated regions (3′-UTR) of SUMO2 gene, which leads to sumoylation of GLUT1 and ubiquitination to regulate the AMPK-mtor pathway, ultimately promoting proliferation and metastasis of nasopharyngeal carcinoma (NPC) (Mo et al., 2021). Circ-RHOBTB3 expression is reduced in colorectal cancer tissues, and lower circ-RHOBTB3 levels are significantly associated with advanced clinical stage and greater risk of metastasis. Circ-RHOBTB3 binds to hur to promote β-Trcp1-mediated hur ubiquitination, which in turn inhibits the invasive effects of CRC (Chen J. et al., 2021).

### Involved in Intercellular Communication Through Exosomes

The main function of exosomes is to mediate intercellular communication through their contents under physiological and pathological conditions (Wang J. et al., 2021; Chen Q. et al., 2021; Shahzad et al., 2021). In addition, exosomes play a role in coagulation, antigen presentation, immune regulation, and viral replication. Exosome contents, such as proteins, mRNAs, and miRNAs, have been shown to act in receptor cells, thereby activating multiple signaling pathways (Kalluri and LeBleu, 2020; Wen et al., 2020; Rong et al., 2021b; Wang et al., 2021e; Reese and Dhayat, 2021; Tian et al., 2021). Tumor cells can secrete more exosomes than normal cells with some variation in contents, and tumor cell-derived exosomes can provide a suitable microenvironment for tumor development, such as cell proliferation, angiogenesis and metastasis, drug resistance and formation of pre-metastatic microenvironment (Li and Wang, 2017; Yan and Chen, 2020). It has been shown that circRNAs can be encapsulated into exosomes and thus participate in tumorigenesis and progression. Exosome-derived hsa_circ_0000337 accelerates Chemoresistance resistance in esophageal cancer cells by regulating the miR-377-3p/JAK2 axis (Zang et al., 2021). Plasma exosomes from colorectal cancer (CRC) patients are enriched in circ-133. Exosomes circ-133 from hypoxic cells are transmitted to normoxic cells and promote CRC metastasis by acting on the miR-133a/GEF-H1/rhoa axis (Yang H. et al., 2020).

### Peptide Translation

Normally circRNAs cannot be flipped, but with deeper research, it was found that exon sequences of some circRNAs can be translated into proteins (Chen and Shan, 2021; Dodbele et al., 2021). Some circRNAs contain internal ribosome entry site (IRES) sequences and can bind directly to ribosomes and can be translated in eukaryotic cells (Abe et al., 2015; He L. et al., 2021). The 40S subunit of eukaryotic ribosomes binds to circRNAs and can directly initiate translation (Wang and Wang, 2015). CircRNAs can also be efficiently translated in an *E. Coli* cell-free translation system with an open reading flame (ORF) (Thompson, 2012). It has also been shown that eukaryotic endogenous circRNAs can drive protein translation through m6a methylation (Yang et al., 2017; Dai et al., 2020).

### Regulation of Gene Expression

CircRNAs can interact with RNA to participate in post-transcriptional regulation. CircRNAs are formed with a balance between competitive complementary pairing between introns and linear RNAs, which affects mrna expression and translation (Shao et al., 2021). The ORF-containing circRNAs produced by COL6A3 encodes a novel 198-aa functional peptide, and hsa_circ_0006401–198-aa promotes the stability of the host gene COL6A3 mrna, thereby facilitating CRC proliferation and translocation (Zhang et al., 2021c). Circ-PTEN can promote CRC proliferation and translocation by acting as a miR-155 Circ-PTEN can increase the expression of its host gene PTEN by acting as a sponge for miR-155 and miR-330-3p, which in turn regulates the PI3K/AKT signaling pathway (Wang et al., 2021i). Regulation of parent genes through RNA polymerase II and epigenetic modifications. Some intron derived circRNAs are mainly localized in the nucleus and can interact with RNA polymerase II to promote transcription of their own coding genes (Li et al., 2015). CircRNAs can also regulate parent gene expression through epigenetic modifications. Recently, some circRNAs were also found to have m6a modifications, which affect the stability of the parent gene (Zhou et al., 2017). Thus, circRNAs can be used to regulate the transcription of disease-related parent genes, which in turn affect the expression of the parent gene and its target genes, providing new ideas for the treatment of corresponding diseases.

## Biological Function and Molecular Mechanism of Circular RNAs in Glioblastoma

Studies have shown that circRNAs have important roles in a variety of tumors, and they can be involved in tumorigenesis and progression through many different mechanisms and are closely associated with the clinical features of tumors (Goodall and Wickramasinghe, 2020). Here, we briefly summarize the circRNAs involved in GBM tumorigenesis and progression and analyze their correlation with the clinical features of GBM.

### Circular RNAs are associated with Proliferation in Glioblastoma

CircRNAs can regulate the cell proliferation ability of GBM by regulating gene expression or downstream signaling pathways (Table 1). A total of 417 aberrantly expressed circRNAs were found in GBM tissues compared with adjacent normal brains by second-generation sequencing, with hsa_circ_0008344 being the most differentially expressed. Overexpression of hsa_circ_0008344 significantly promoted proliferation, colony formation and decreased apoptosis in GBM cells (Zhou et al., 2018). Eukaryotic initiation factor 4A3 (EIF4A3) bound to MMP9 mrna transcripts induced circ-MMP9 cyclization and promoted circ-MMP9 expression in GBM. MMP9 promotes the proliferative capacity of GBM cells by targeting miR-124 to regulate the expression of CDK4 and AURKA (Wang et al., 2018b). Hsa_circ_0074027 expression is significantly upregulated in GBM and is associated with clinical features. Hsa_circ_0074027 promotes IL17RD expression through sponge binding of miR-518a-5p, which in turn promotes the proliferative capacity of cells (Qian et al., 2019). Circ-PITX1 enhances MAP3K2 expression by binding miR-379-5p as a competitive endogenous RNA (ceRNA) sponge, which promotes cell proliferation and inhibits apoptosis in GBM(Lv et al., 2019). In addition, Cao et al.,. Also found that circ-PITX1 was significantly overexpressed in GBM tissues and cells, and knockdown of circpitx1 inhibited cell proliferation and tumor growth. Circ-PITX1/miR-584-5p/KPNB1 axis may be a potential therapeutic target for GBM (Cao et al., 2021). Hsa_circ_0001801 upregulates HMGB3 expression in GBM through sponge binding of miR-628-5p, thereby promoting cell proliferation (Chen et al., 2019). Zhu et al., Found that circentpd7 (circbase ID:hsa_circ_0019421) was upregulated in GBM tissues, and knockdown of circentpd7 significantly inhibited GBM cell motility and proliferation (Zhu et al., 2020). Hsa_circ-0043278-miR-638/-HOXA9 regulatory axis has an important role in GBM progression by regulating miR-638/- HOXA9. Hsa_circ-0043278-miR-638/- HOXA9 regulatory axis has an important role in GBM progression and can be involved in GBM tumorigenesis and progression by regulating cell proliferation (Wu et al., 2020). Circ-ABCC3 acts as a sponge for miR-770-5p, which targets SOX2, and knockdown of Circ-ABCC3 significantly inhibits tumor growth *in vivo* (Zhang and Xu, 2021). Wang et al., Found that hsa_circ_0006168 may promote tumor growth in GBM by acting as a competitive endogenous RNA for miR-628-5p and regulating the IGF1R/Ras/Erk pathway (Wang T. et al., 2021). Circ_0001588 may promote the proliferative capacity of GBM cells by regulating the miR-211-5p/YY1 signaling pathway (Wang Q. et al., 2021).

**TABLE 1:** The proliferation-related circular RNAs in GBM.

circRNAs	Expression	Mechanism	Biological function	Ref.PMID
hsa_circ_0008344	Up	—	Promote cell proliferation, colony formation and inhibit cell apoptotic rate	29687495
circ-MMP9	Up	miR-124/CDK4	Promote cell proliferation	30470262
hsa_circ_0029426	Up	miR-197	Promote cell proliferation and inhibit cell apoptosis	30548670
hsa_circ_0074027	Up	miR-518a-5p/IL17RD	Promote cell proliferation, colony formation and inhibit cell apoptotic rate	30738578
hsa_circ_0067934	Up	PI3K-AKT	Promote cell proliferation	31081099
circ-PITX1	Up	miR-379–5p/MAP3K2	Promote cell proliferation and inhibit cell apoptosis	31493405
circ-FOXO3	Up	miR-138-5p/miR-432-5p/NFAT5	Promote cell proliferation	31504797
hsa_circ_0001801	Up	miR-628-5p/HMGB3	Promote cell proliferation	31858556
circ-EPB41L5	Up	miR-19a/EPB41L5	Promote cell proliferation and colony formation	31905344
circ-ENTPD7	Up	miR-101-3p/ROS1	Promote cell proliferation	32308563
hsa_circ_0043278	Up	miR-638/HOXA9	Promote cell proliferation	33154193
circ-SMO	Up	SMO-193aa	Promote cell proliferation	33446260
circ-SKA3	Up	miR-1	Promote cell proliferation	33500664
circ-PARP4	Up	miR-125a-5p	Promote cell proliferation	33520365
circ-PITX1	Up	miR-584-5p/KPNB1	Promote cell proliferation	33763840
circ-ABCC3	Up	miR-770-5p/SOX2	Promote cell proliferation and inhibit cell apoptosis	33811842
hsa_circ_0006168	Up	miR-628-5p/IGF1R	Promote cell proliferation, colony formation and inhibit cell apoptotic rate	34024251
hsa_circ_0001588	Up	miR-211-5p/YY1	Promote cell proliferation	34105224
circ-FLN1	Up	miR-199-3p	Promote cell proliferation	34498720
circ-SERPINE2	Up	miR-361-3p/miR-324-5p/BCL2	Promote cell proliferation, colony formation and inhibit cell apoptotic rate	34553034
	—	—		—
circ-LGMN	Up	miR-127-3p/LGMN	Promote cell proliferation	34582975
circ-NF1	Up	miR-340	Promote cell proliferation	34589042
circ-NT5E	Down	miR-422a	Inhibit cell proliferation	29967262
hsa_circ_0001946	Down	miR-671-5p/CDR1	Inhibit cell proliferation and promote cell apoptosis	30663767
circ-MTO1	Down	miR-92/WWOX	Inhibit cell proliferation	31456594
circ-AKT3	Down	AKT3-174aa/PI3K/AKT	Inhibit cell proliferation	31470874
hsa_circ_01844	Down	—	Inhibit cell proliferation and promote cell apoptosis	32804726
circ-CDR1as	Down	p53/MDM2	Inhibit cell proliferation	32894144

Moreover, circRNAs can also be involved in regulating cell proliferation by binding multiple miRNAs to regulate the expression of downstream genes. Found that circ-FOXO3 can bind both miR-138-5p and miR-432-5p to regulate NFAT5 expression and thus promote cell proliferation (Zhang S. et al., 2019). Li et al., Found that the circ-SERPINE2-miR-361-3p/miR-324-5p/BCL2 signaling pathway plays an important role in the tumor growth process of GBM (Li D. et al., 2021). Besides, hsa_circ_0008344 (Zhou et al., 2018), hsa_circ_0029426 (Zhang G. et al., 2019), hsa_circ_0067934 (Xin et al., 2019), circ-SKA3 (Zhou M. et al., 2021), circ-PARP4 (Zhou J. et al., 2021), circ-FLN1 (Sun et al., 2021) and circ-NF1(Liu L. et al., 2021) were also shown to significantly promote the proliferative capacity and tumor growth of GBM cells, but the specific molecular mechanisms remain to be further explored.

Hsa_circ_0001946 inhibits cell proliferation and promotes apoptosis by binding to miR-671-5p and regulating CDR1 expression (Li and Diao, 2019). Circ-MTO1 inhibits tumor growth of GBM through miR-92/WWOX regulatory axis (Zhang X. et al., 2019). Circ-NT5E inhibits cell proliferation of GBM by binding miR-422a (Wang et al., 2018a). Circ-CDR1as regulates the malignant growth of GBM by modulating the p53/MDM2 signaling pathway (Lou et al., 2020).

In recent years, circRNAs have been found to be involved in disease processes by encoding peptides. Found that circ-SMO encodes a peptide of length 193aa SMO-199aa. Knockdown deprivation of SMO-193aa in GBM stem cells significantly attenuated Hedgehog signaling and inhibited self-renewal, proliferation *in vitro* and tumorigenicity *in vivo* (Wu X. et al., 2021). Xia et al., Found that circ-AKT3 encodes a novel 174 aa protein, AKT3-174aa, and overexpression of AKT3-174aa significantly reduced cell proliferation, radioresistance and tumorigenicity of GBM cells *in vivo*, while overexpression of circ-AKT3 suppressed the malignant phenotype of GBM(Xia et al., 2019).

### Circular RNAs are associated with Invasion and Metastasis in Glioblastoma

Invasion and metastasis of tumor cells are the main cause of death in most patients with malignant tumors (Asif et al., 2021). This ability to invade and metastasize allows tumor cells to leave their primary location within tissues, enter lymphatic vessels and blood vessels, and colonize distant organs with the blood circulation. Metastasis of tumor cells is a complex, dynamic process that occurs through cytoskeletal remodeling to form leading edge protrusions, thereby generating mechanical forces that retract and separate the cell tails from the extracellular matrix. CircRNAs have vital roles in the invasion and metastasis of GBM. Hsa_circ_0008344 (Zhou et al., 2018), circ-MMP9(Wang et al., 2018b), hsa_circ_0029426 (Zhang G. et al., 2019), hsa_circ_0074027 (Qian et al., 2019), hsa_circ_0067934 (Xin et al., 2019), circ-FOXO3 (Zhang S. et al., 2019), hsa_circ_0001801 (Chen et al., 2019), circ-EPB41L5 (Lv et al., 2020), circ-ENTPD7 (Zhu et al., 2020), hsa_circ_0043278 (Wu et al., 2020), circ-PARP4 (Zhou J. et al., 2021), circ-SMARCA5 (Barbagallo et al., 2021), circ-PITX1 (Cao et al., 2021), circ-ABCC3(Zhang and Xu, 2021), hsa_circ_0006168 (Wang T. et al., 2021), hsa_circ_0001588 (Wang Q. et al., 2021), circ-MELK (Zhou F. et al., 2021), circ-FLN1 (Sun et al., 2021), circ-LGMN(Chen B. et al., 2021), circ-NT5E (Wang et al., 2018a), hsa_circ_0001946 (Li and Diao, 2019) and circ-MTO1 (Zhang X. et al., 2019) are involved in the regulation of GBM invasion and metastasis (Table 2).

**TABLE 2:** The migration and invasion-related circular RNAs in GBM.

circRNAs	Expression	Mechanism	Biological function	Ref.PMID
hsa_circ_0008344	Up	—	Promote cell migration and invasion	29687495
circ-MMP9	Up	miR-124/CDK4	Promote cell migration and invasion	30470262
hsa_circ_0029426	Up	miR-197	Promote cell migration and invasion	30548670
hsa_circ_0074027	Up	miR-518a-5p/IL17RD	Promote cell migration and invasion	30738578
hsa_circ_0067934	Up	PI3K-AKT	Promote cell migration and invasion	31081099
circ-FOXO3	Up	miR-138-5p/miR-432-5p/NFAT5	Promote cell migration and invasion	31504797
hsa_circ_0001801	Up	miR-628-5p/HMGB3	Promote cell migration and invasion	31858556
circ-EPB41L5	Up	miR-19a/EPB41L5	Promote cell migration and invasion	31905344
circ-ENTPD7	Up	miR-101-3p/ROS1	Promote cell migration and invasion	32308563
hsa_circ_0043278	Up	miR-638/HOXA9	Promote cell migration and invasion	33154193
circ-PARP4	Up	miR-125a-5p	Promote cell migration and invasion	33520365
circ-SMARCA5	Up	—	Promote cell migration and invasion	33562358
circ-PITX1	Up	miR-584-5p/KPNB1	Promote cell migration and invasion	33763840
circ-ABCC3	Up	miR-770-5p/SOX2	Promote cell migration and invasion	33811842
hsa_circ_0006168	Up	miR-628-5p/IGF1R	Promote cell migration and invasion	34024251
hsa_circ_0001588	Up	miR-211-5p/YY1	Promote cell migration and invasion	34105224
circ-MELK	Up	miR-593/EphB2	Promote cell migration and invasion	34168916
circ-FLN1	Up	miR-199-3p	Promote cell migration and invasion	34498720
circ-LGMN	Up	miR-127-3p/LGMN	Promote cell migration and invasion	34582975
circ-NT5E	Down	miR-422a	Inhibit cell migration and invasion	29967262
hsa_circ_0001946	Down	miR-671-5p/CDR1	Inhibit cell migration and invasion	30663767
circ-MTO1	Down	miR-92/WWOX	Inhibit cell migration and invasion	31456594

Epithelial-mesenchymal transition (EMT) is mainly involved in embryogenesis, organogenesis, and tissue healing in humans, but also in tumorigenesis and metastasis, promoting tumor cell invasion and motility by altering intercellular interactions and cell-matrix interactions (Inoue et al., 2015; Wu N. et al., 2021; Satcher and Zhang, 2021; Xiong et al., 2021). About 90% of tumor patient deaths result from tumor invasion and metastasis, which suggests that regulation of the EMT process is important for tumor prevention and treatment. E⁃cadherin expression is suppressed upon EMT activation, resulting in the loss of the typical polygonal cobblestone morphology of epithelial cells, while cells acquire a spindle-shaped mesenchymal morphology and express markers associated with the mesenchymal cell state, particularly N-cadherin, wave proteins and fibronectin (Cristofanilli and Mendelsohn, 2006; Cai et al., 2021; Zhang N. et al., 2021), therefore, the activation status of EMT can be assessed by changes in the expression of E⁃cadherin and N-cadherin. It has been shown that growth factors such as epidermal growth factor (EGF), transcription factors such as Snail, Slug, Twist, E⁃box binding zinc finger protein (ZEB) and signaling pathways such as TGF, Wnt, Notch and Hedgehog can mediate the EMT. Mucin (MUC) acts as an inducer to activate various signaling pathways that also contribute to EMT (Ponnusamy et al., 2013). We found that among the circRNAs involved in GBM, circ-MMP9(Wang et al., 2018b), hsa_circ_0067934 (Xin et al., 2019), hsa_circ_0001801 (Chen et al., 2019), circ-PARP4 (Zhou J. et al., 2021), circ-PITX1 (Cao et al., 2021), hsa_circ_0006168 (Wang T. et al., 2021), hsa_circ_0001588 (Wang Q. et al., 2021) and circ-MELK (Zhou F. et al., 2021) can regulate GBM invasion and metastasis by modulating the EMT process. Hsa_circ_0067934 (Xin et al., 2019), circ-EPB41L5 (Lv et al., 2020) and circ-ABCC3(Zhang and Xu, 2021) can participate in the invasion and metastasis of GBM by regulating the PI3K/Akt/mtor signaling pathway.

### Circular RNAs are associated with angiogenesis in Glioblastoma

**FIGURE 3:**
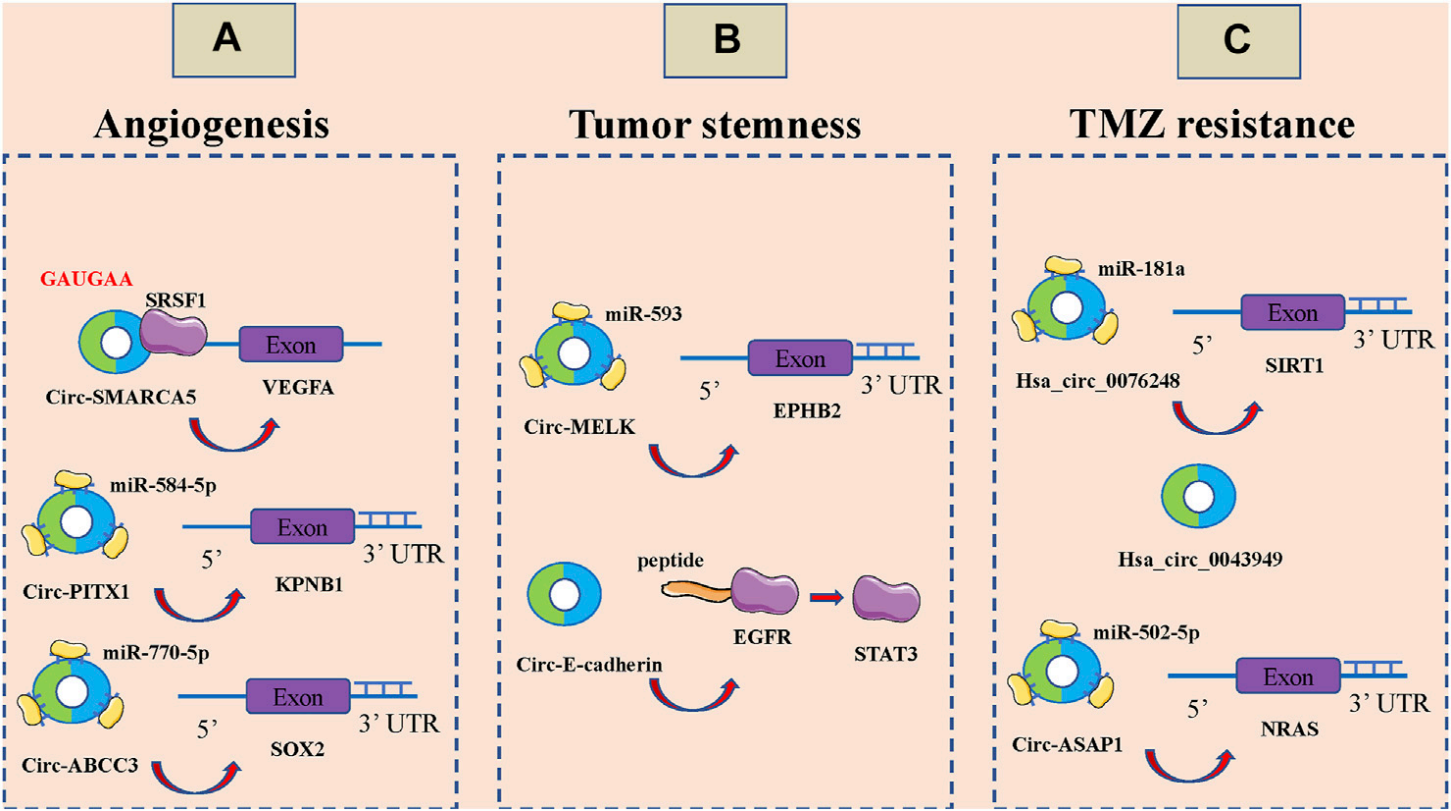
CircRNAs associated with the angiogenesis, tumor stemness and temozolomide (TMZ) resistance of glioblastoma (GBM). **(A)**. CircRNAs associated with the angiogenesis of GBM, including circ-SMARCA5, circ-PITX1 and circ-ABCC3. **(B)**. CircRNAs associated with the tumor stemness of GBM, including circ-MELK and circ-E-cadherin. **(C)**: CircRNAs associated with the temozolomide (TMZ) resistance of glioblastoma GBM, including hsa_circ_0076248, hsa_circ_0043949 and circ-ASAP1.

Tumor development, invasion and metastasis are highly dependent on neovascularization (Goncalves et al., 2021; Rimini and Casadei-Gardini, 2021). Under physiological conditions, angiogenesis is intricately and precisely regulated by multiple molecules and mechanisms that allow the formation of highly tissue-specific, structured and hierarchical vascular networks to sustain the physiological processes of embryonic development, growth and tissue repair (Lai et al., 2021; Martin and Gurevich, 2021; Narasimhan et al., 2021). However, tumor cells are able to release large amounts of vascular endothelial growth factor (VEGF) and inhibit the secretion of angiogenesis inhibitory factor, which unbalances the regulatory mechanism of angiogenesis, resulting in rapid, uncontrolled proliferation of tumor neovascularization and a large, abnormally disordered replenishment network (Liu Y. et al., 2021; Uemura et al., 2021; Zhu et al., 2021). CircRNAs can also be involved in the malignant invasion and metastasis of GBM by affecting angiogenesis (Figure 3A). Barbagallo et al., Found that circ-SMARCA5 regulates VEGFA mrna Splicing and angiogenesis through binding to SRSF1, which in turn leads to malignant progression of GBM(Barbagallo et al., 2019). In addition, they demonstrated that the GAUGAA motif is a key sequence for binding of circ-SMARCA5 to SRSF1(Barbagallo et al., 2021). Cao et al., The circ-PITX1/miR-584-5p/KPNB1 regulatory axis was found to be an important molecular mechanism mediating GBM angiogenesis (Cao et al., 2021). Circ-ABCC3 regulates GBM angiogenesis and tumor malignancy progression through PI3K/AKT signaling pathway and miR-770-5p/SOX2 axis (Zhang and Xu, 2021).

### Circular RNAs are associated with Tumor Stemness in Glioblastoma

Stem cells are widely involved in body growth and development and organ formation, and have the ability of self-renewal, infinite proliferation and multidirectional differentiation (Jing et al., 2021; Mehraj et al., 2021; Mirzadeh Azad et al., 2021; Otero-Albiol and Carnero, 2021). The strong self-renewal ability, inherent high proliferative capacity and multidirectional differentiation together constitute the basic characteristics of malignant stem cells, among which the self-renewal ability is closely related to tumorigenesis and malignancy (Ferragut et al., 2021; Mehraj et al., 2021; Yue et al., 2021). Over the past three to 4 decades, numerous studies have noted a potential link between stem cell systems and certain tumors, and a small proportion of tumor-initiating cells with stem cell properties, also known as tumor stem cells, have been identified in a variety of organs (Cermeno and Garcia, 2016; Hass et al., 2020; Pan et al., 2021; Ryskalin et al., 2021). Tumor stem cells of glioma tissue origin have the capacity for self-renewal, homotransplantation into tumors, and differentiation into neurons and glial cells; it is now thought that they may be derived from genetically mutated neural stem cells, transiently expanded cells, neural progenitor cells, and even highly differentiated astrocytes and oligodendrocytes in normal brain tissue. It has been found that circRNAs also play an important role in GBM cell stemness (Figure 3B). Zhou et al., Found that circ-MELK expression was significantly increased in GBM tissues and that circ-MELK could regulate GBMEMT progression and glioma stem cells (GSCs) maintenance by binding to miR-593 to promote ephb2 expression (Zhou F. et al., 2021). Gao et al., Demonstrated that circ-E-cadherin encodes a 14 amino acid peptide that binds to the CR2 structural domain of EGFR and activates EGFR-STAT3 signalling, thereby maintaining the tumorigenicity of glioma stem cells (Gao et al., 2021).

### Circular RNAs are associated with Temozolomide Resistance in Glioblastoma

TMZ is an alkylating agent with nearly 100% oral bioavailability and easily crosses the blood-brain barrier, and is currently the first-line chemotherapeutic agent for the treatment of GBM(Al-Toubah et al., 2021; Li F. et al., 2021; Soni et al., 2021). The breakdown products of TMZ can cause DNA methylation after entering tumor cells, which can interfere with cellular DNA replication and cause DNA damage to inhibit the proliferation of tumor cells (Tomar et al., 2021; Winkler, 2021). However, there is a strong DNA damage repair system and complex damage repair mechanisms in GBM cells, which are important in mediating the development of resistance to TMZ in GBM (Trillo Aliaga et al., 2021; Zhang X.-N. et al., 2021). It has been shown that circRNAs are also involved in TMZ resistance of GBM (Figure 3C). Lei et al., Hsa_circ_0076248 was found to be involved in the malignant progression of glioma by binding miR-181a to promote SIRT1 expression, and upregulation of hsa_circ_0076248 significantly inhibited temozolomide chemotherapy sensitivity (Lei et al., 2019). Zhao et al., The expression profiles of circRNAs in three pairs of secondary temozolomide-resistant GBM and the corresponding primary GBM tissues were examined by microarray. The high expression of hsa_circ_0043949 was found to be closely associated with the resistance of TMZ (Zhao et al., 2020). Wei et al., Inhibition of circ-ASAP1 was found to be effective in restoring the sensitivity of TMZ-resistant xenografts to TMZ treatment *in vivo*, possibly by regulating NRAS expression through binding to miR-502-5p (Wei et al., 2021).

### Relationships Between Circular RNAs Levels and Clinicopathologic Characteristics in Glioblastoma

Studies have shown that the expression levels of circRNAs significantly correlate with many clinicopathological features of GBM, including tumor size, grading, differentiation and staging, and tumor recurrence. Found that the expression of hsa_circ_0029426 was correlated with tumor size and WHO classification (Zhang G. et al., 2019). The expression of hsa_circ_0074027 was found to be closely associated with larger tumor size and higher WHO grade (Qian et al., 2019). It was found that high levels of circ-ENTPD7 correlated with advanced GBM classification and tumor size (Zhu et al., 2020). Sun et al., Demonstrated that the expression level of circ-FLNA correlated significantly with the presence of necrosis in MRI scans (Sun et al., 2021) (34498720). Liu et al., A multivariate Cox regression analysis revealed that circnf1 expression was an independent prognostic factor for GBM patients (Liu L. et al., 2021). Lv et al., The analysis found that the expression of circ-EPB41L5 correlated with age, number of lesions, necrotic changes, recurrence and survival in GBM patients (Lv et al., 2020). Wang et al., found that hsa_circ_0006168 expression significantly correlated with WHO classification, T-stage, N-stage and M-stage (Wang T. et al., 2021).

### Circular RNAs as Prognostic Biomarkers for Glioblastoma

The expression levels of circRNAs were found to be used to predict the prognosis of tumor patients. To further analyze the prognostic value of circRNAs in GBM, we evaluated the association of circRNAs expression levels with the overall survival (OS) rate of GBM patients. Twelve upregulated circRNAs were reported to predict poorer OS in GBM patients (Zhang G. et al., 2019; Qian et al., 2019; Xin et al., 2019; Zhu et al., 2020; Chen B. et al., 2021; Li D. et al., 2021; Liu L. et al., 2021; Wang Q. et al., 2021; Wu X. et al., 2021; Zhou M. et al., 2021; Sun et al., 2021), and three downregulated circRNAs predicted poorer OS in GBM patients (Zhang X. et al., 2019; Lv et al., 2020; Wang T. et al., 2021). It was shown that lower expression of has_circ_0067934 was associated with longer disease-free survival (DFS) (Xin et al., 2019). Higher expression of circ-EPB41L5 was associated with longer progress-free survival (PFS) (Lv et al., 2020). Kaplan-Meier analysis showed that GBM patients with low circ-ASAP1 expression showed better OS after TMZ treatment compared to GBM patients with high circ-ASAP1 expression (Wei et al., 2021) (Table 3).

**TABLE 3:** Utility of circRNAs for clinical of GBM.

circRNAs	Expression	Clinical Sample	Diagnostic	Utility Prognostic	Predictive	Ref.PMID
has_circ_0029426	Up	Tissues	—	√	√	30548670
has_circ_0074027	Up	Tissues	—	—	√	30738578
has_circ_0067934	Up	Tissues	—	√	—	31081099
circ-ENTPD7	Up	Tissues	—	√	√	32308563
circ-SMO	Up	Tissues	—	√	—	33446260
circSKA3	Up	Tissues	—	√	—	33500664
hsa_circ_0001588	Up	Tissues	—	√	—	34105224
circ-FLNA	Up	Tissues	—	√	√	34498720
circ-SERPINE2	Up	Tissues	—	√	—	34553034
circ-LGMN	Up	Tissues	—	√	—	34582975
circ-NF1	Up	Tissues	—	√	√	34589042
circ-ASAP1	Up	Tissues	—	√	—	32,926,734
circ-MTO1	Down	Tissues	—	√	—	31456594
circ-EPB41L5	Down	Tissues	—	√	√	31905344
hsa_circ_0006168	Down	Tissues	—	—	√	34024251

## Conclusion and Future Prospects

CircRNAs are highly stable, richly expressed, and functionally diverse, and have begun to attract the attention of researchers in recent years, but research on circRNAs is currently in its infancy (Ducoli and Detmar, 2021; Winkle et al., 2021). Nevertheless, the almost complete sequence overlap between circRNAs and linear RNAs makes the accurate assessment of the expression and function of circRNAs still challenging (Yang X. et al., 2020; Jusic et al., 2020). For example, if exonic circRNAs are formed by reverse splicing, how does the spliceosome specifically recognize the exons of circRNAs, but not those of linear RNAs. Recent studies have found that m6a-modified circRNAs are usually derived from exons that are not methylated in mRNAs, and circRNAs from methylated mrna exons are less stable, and it is still unclear whether m6a modification affects the stability of circRNAs. CircRNAs are degraded, and the loop structure may confer different properties to their corresponding linear RNAs. The functional implications of circRNAs have only been tentatively explored, probably due to the limitations of the research tools.

Recent studies on circRNAs have focused on their miRNAs sponge function, researchers have verified the binding sites of circRNAs and miRNAs by luciferase reporter system, pull-down experiments using biotin-labeled probes to capture miRNAs, Ago2 immunoprecipitation can also further investigate miRNAs regulatory targets, co-localization of circRNAs and miRNAs in cells can be verified using FISH technique, and these mature experimental techniques now demonstrate the miRNAs sponge function of circRNAs, making the whole cerna network more complete and complex.

Nowadays, there are more and more researches on the mechanism of circRNAs generation. In addition to RBP can bind to circRNAs to reduce the regulation of RBP on target genes and affect tumorigenesis (Huang C.-K. et al., 2020; Li et al., 2020; Yang J. et al., 2021; Song et al., 2021), some RBP can also regulate the generation of circRNAs, which also has an important role in tumor development (2016; Li H. et al., 2021; Liu Z. et al., 2021; Wang et al., 2021d; Wang and Lei, 2021; Yang T. et al., 2021). The translation function of circRNAs is also becoming a hot topic, and the translation initiation mechanism of circRNAs mainly includes cap-dependent, IRES-dependent, m6a-dependent and small ORF-dependent translation initiation (He L. et al., 2021; Wang X. et al., 2021), and circRNAs encode proteins with the function of suppressing tumor activity and protecting proteins from degradation (Chen C.-K. et al., 2021; Wang et al., 2021d; Ma et al., 2021; Qi et al., 2021). In summary, the interactions between circRNAs and proteins and its translation function are of great significance for the study of tumors, and more in-depth studies should be conducted in this area in the future.

CircRNAs are highly stable and widely expressed in a variety of tissues and body fluids, and thus can be used as potential diagnostic and prognostic biomarkers. Studies have shown that circRNAs are abundant and stable in exosomes, and some circRNAs are more highly expressed in blood than in tissues (D'Ambrosi et al., 2021; Reese and Dhayat, 2021; Tian et al., 2021; van Zonneveld et al., 2021). The high expression and stability of circRNAs in body fluids would be more beneficial for their clinical applications (Bu et al., 2021; Fontemaggi et al., 2021; Wang et al., 2021h; Zhao et al., 2021). However, despite the identification of thousands of tissue- and disease-specific circRNAs by RNA-seq (Kaushik et al., 2021; Tian-Zhao et al., 2021), the understanding of the mechanism of circRNAs generation and biological functions is limited at this stage. Further research is needed to explore the circRNAs associated with GBM, with the aim of being able to be used in combination with traditional biological diagnostic indicators for clinical adjuvant screening of GBM at an early stage, thus indirectly improving the survival rate of GBM patients and achieving early detection and treatment. It is believed that as more and more GBM-related and structurally diverse circRNAs are discovered, the elucidation of complex molecular regulatory mechanisms of GBM and the application of circRNAs-based GBM diagnosis and treatment will have a broad prospect.

In summary, circRNAs are important regulators of GBM genesis and can act as endogenous RNAs or miRNAs sponges competitively repressing miRNAs, thereby altering target gene expression and participating in the development of GBM. Although the exact role of circRNAs in GBM genesis and prognosis is unknown, it is speculated that aberrant expression of circRNAs and their biased distribution in tumors may be common, with different circRNAs expressed up- or down-regulated in GBM cells and tissues, acting through different mechanisms of action.

## Data Availability

The data in the current study are available from the corresponding authors on reasonable request.

## Abbreviations

CircRNAs, circular RNAs; GBM, glioblastoma; CNS, central nervous system; Exonic circRNAs, E-circRNAs; ciRNAs, intron circRNAs; eiciRNAs, exon-intron circRNAs; miRNAs, microRNAs; NSCLC, non-small-cell lung cancer; EMT, epithelial mesenchymal transition; PDAC, pancreatic ductal adenocarcinoma; RBP, RNA binding protein; 3′-UTR, 3′ untranslated regions; NPC, nasopharyngeal carcinoma; CRC, colorectal cancer; IRES, internal ribosome entry site; ORF, open reading flame; EIF4A3, Eukaryotic initiation factor 4A3; ceRNA, competitive endogenous RNA; EDF, epidermal growth factor; E⁃box binding zinc finger protein, ZEB; MUc, Mucin; VEGF, vascular endothelial growth factor; GSCs, glioma stem cells; TMZ, temozolomide; OS, overall survival; DFS, disease-free survival; PFS, progress-free survival.
